# Effect of vaccine storage temperatures and dose rate on antibody responses to foot and mouth disease vaccination in Cambodia

**DOI:** 10.1002/vms3.86

**Published:** 2017-11-29

**Authors:** Socheat Sieng, Stephen W. Walkden‐Brown, James Kerr

**Affiliations:** ^1^ School of Behavioural, Cognitive and Social Sciences University of New England Armidale New South Wales Australia; ^2^ General Directorate of Animal Health and Production Phnom Penh Cambodia; ^3^ Animal Science School of Environmental and Rural Science University of New England Armidale New South Wales Australia

**Keywords:** farmer, foot and mouth disease, cattle, Vaccine, ELISA, Cambodia

## Abstract

A field study investigated the effects of foot and mouth disease vaccine storage temperature for 7 days (frozen, refrigerated or held at ambient temperature) and dose (half or full dose) on the serological response to vaccination. It utilised a complete factorial design replicated on 18 smallholder cattle farms in three villages in Pursat province, Cambodia. Antibody responses from the 108 cattle involved were assessed by serological examination of blood samples collected at primary vaccination (day 0), at booster vaccination (day 30) and finally at 60 days post primary vaccination. Vaccination responses to the inactivated vaccine were assessed by testing for antibodies directed against FMD structural proteins in a liquid‐phase blocking ELISA (LPBE test) and differentiated from responses to natural infection by examining antibody titres against non‐structural viral proteins (NSPE test). LPBE results indicated that the mean log_10_ LPBE antibody titres of all experimental cattle increased from below protective levels at day 0 to protective levels at 30 days post primary vaccination, and increased further at 60 days post primary vaccination. Storage at ambient temperature for 1 week had no effect on antibody response to vaccination. However, freezing the vaccine for a week or use of a half dose resulted in significant reduction in titres at day 60 (*P* = 0.04 and *P* = 0.02, respectively). The results of this study reinforce the need to store FMD vaccines within the range recommended by the manufacturers and to adhere to the specified dosage instructions.

## Introduction

Foot and mouth Disease (FMD) is one of the most important livestock diseases affecting both domestic and wild cloven‐hoofed animals including cattle, buffalo, sheep, goats and pigs (Eblé *et al*. 2004; Alexandersen & Mowat 2005). Its importance lies mostly in its extreme contagiousness with FMD being one of the most contagious animal diseases known (Doel *et al*. 1994). FMD can be spread by direct and indirect contact with infected animals, animal products or contaminated materials. This disease is still endemic in six Southeast Asian countries including Cambodia, Laos PDR, Malaysia, Myanmar, Thailand and Vietnam. Malaysia is in a progressing towards becoming an OIE recognised FMD free zone. Brunei, Indonesia, Philippines and Singapore have been recognised as FMD free countries where vaccination is not practised by OIE since 2011. Three serotypes of FMDV (A, O and Asia 1) are common in the SEA region with the predominant strain being serotype O. Several topotypes are reported and the Cathay topotype appears predominant (Gleeson & Ozawa 2002; Khounsy *et al*. 2009). Over the last 10 years, serotype O and Asia 1 have been detected in field outbreaks in Cambodia, but serotype A has not been reported. Even though the disease does not result in high mortality in most outbreaks, it has a significant impact on livestock systems in Cambodia due to reduced meat and milk production, temporary loss of draught capacity and losses from reduced trade (Kazimi & Shah 1980; Morris *et al*. 2002; Perry *et al*. 2002). Several studies in Cambodia and Laos have shown that the impact of FMD on farmers is high due to the reduction of cattle value (weight loss, treatment cost, draught replacement) at the household and village levels (Young *et al*. 2012; Nampanya *et al*. 2013, 2015).

Different approaches and measures have been adopted to control the spread of FMD in many parts of the world. These include vaccination, movement restrictions and other biosecurity measures, destruction of infected animals and surveillance (Keeling *et al*. 2002). For efficient control of FMD where it is endemic, vaccination every 6 months and restriction of the movement of sick animals and their products is crucial (Parida 2009). Studies have revealed that effective vaccination is an important FMD control measure in these situations (Hunter 1998; Parida 2009) and can stop or reduce the spread of FMD from infected to vaccinated animals, and from affected to unaffected areas (Hunter 1998; Khounsy *et al*. 2008; Parida 2009). Killed or purified antigen monovalent, bivalent, trivalent or polyvalent FMD are produced by more than twenty companies around the world (CFSPH 2004) incorporating oil or aluminium hydroxide based adjuvants. In Cambodia, the vaccines used are Aftopor^®^ Trivalent, Oil Adjuvanted, Inactivated Purified Vaccine against Foot and Mouth Disease (virus strains: O_Manisa_, O_‐3039_, AMay 97, Asia‐1, Merial), Aftopor^®^ Monovalent, Oil Adjuvant, Inactivated Purified Vaccine against Foot and Mouth Disease (Virus strain: O_Manisa_+O_3039_, Merial) and Raksha‐Ovac Trivalent, Oil Adjuvant, Killed Vaccine against Foot and Mouth Disease (Virus strain: O, A and Asia‐1, Indian Immunologicals limited).

FMD control in Cambodia relies on ring vaccination around outbreaks. The approach taken is varied to suit local circumstances, such as the availability of funds and vaccines for the implementation of a vaccination program. Interestingly, results from a study in one Cambodian province indicated that more than half of cattle vaccinated with donated FMD vaccines subsequently became infected with FMD virus and showed clinical signs of FMD indicating possible vaccine failure (Sieng & Kerr 2013). The possible reasons for such results include poor planning and execution of the vaccination program, vaccine cold chain breakdown and poor vaccination technique. In the dose–response experiment of Brehm *et al*. (2008) using full dose, 1/4 dose and 1/16 dose of high potency FMD vaccines decreasing vaccine dose was associated with a reduced level of protection against FMD virus. A recent study into vaccine storage temperatures in Cambodia revealed that FMD and other vaccines were routinely exposed to temperature outside the recommended minimum and maximum temperatures range in most of the veterinary drugstores investigated (Sieng *et al*. 2016). Another study demonstrated that a type O FMD vaccine maintained 100% efficacy following storage for 2 years at 4°C, for 3 weeks at 25°C and for 1 week at 37°C with protection reduced to 80% after storage at 25°C for 4 weeks or 37°C for 2 weeks (El‐Sayed *et al*. 2012).

We therefore designed the present study to test the broad proposition that inappropriate storage temperatures and/or under dosing would produce measurable reductions in the efficacy of FMD vaccination under Cambodian field conditions. The ultimate test of vaccine efficacy is protection against disease challenge, but this is not always easily measured. Serological responses to vaccination provide an alternative measure of efficacy and are based on the association between anti‐vaccine antibody titre and disease protection. Non‐structural proteins ELISA (NSPE) can be used to detect the NSPs of replicating FMD virus indicating past or present active infection with FMD virus. International standard sera for NSP testing of cattle have been developed and are available from the OIE Reference Laboratory, Panaftosa, PAHO/WHO (OIE, 2012). On the other hand, antibody responses to FMD virus structural proteins are elicited by vaccination with inactivated vaccines and active infection. Such antibody responses can be detected by the virus neutralisation test (VNT), the liquid‐phase blocking ELISA (LPBE, used in this study), or the solid‐phase competition ELISA (SPCE). These tests are internationally accepted and prescribed tests for trade and are appropriate for confirming previous or ongoing infection in non‐vaccinated animals as well as for monitoring the immunity conferred by vaccination in the field (OIE, 2012).

## Materials and methods

### Experimental design

The experiment had a repeated measures design incorporating a complete 3 × 2 factorial arrangement of treatments with measurements at three times. The effects of FMD vaccine storage conditions for 7 days prior to use (freezer, refrigerator, or ambient temperature) and FMD vaccine dose (half or full dose) were replicated on 18 smallholder farms in three villages in Pursat province Cambodia. The six treatments were allocated to individual cattle at random on each smallholder farm with vaccinations administered on day 0 followed by a booster vaccination 30 days later (day 30). Responses to vaccination were assessed serologically by evaluating antibody titres to structural and non‐structural FMD viral proteins at days 30 and 60 relative to the primary vaccination and comparing them with the initial titres obtained on day 0.

### Study area, cattle numbers and records

The experiment started in November 2014 and finished in January 2015 and involved 18 smallholder farmers in three villages of Pursat province (Table 1). To match the six treatments in the experiment, the number of cattle for each participating farmer was restricted to six animals over 6 months of age providing 108 cattle in total for the experiment, with each farm representing a complete experimental replicate. The six treatments were allocated to individual cattle at random on each farm from the first vaccination with animals receiving the same treatment at the booster vaccination on day 30. Cattle were individually identified and prior to each vaccination and/or blood sampling individual animal information including nick name, breed, sex, age, condition score, history of FMD vaccination and other animal health interventions was recorded in addition to any current clinical signs of disease. Date and names of the person who performed vaccinations and blood sample collections were also recorded.

**Table 1:** Details of farmer numbers, cattle numbers and type in the study by village in Roleap Commune, Sampov Meas District, Pursat Province, Cambodia

Village	No. farmers	Total no. cattle	Number of study cattle				
			Heifers	Cows	Steers	Bulls	Total
Prey Ourmal	6	55	9	11	3	13	36
Roleap	6	62	2	19	2	13	36
Or Thkov	6	62	9	12	4	11	36
Total	18	179	20	42	9	37	108

### Preparation of FMD vaccines

A commercial, monovalent inactivated, purified vaccine against foot and mouth disease (Aftopor monovalent, virus strain O_manisa_+O_3039_, batch no. 0‐404, manufacture date 31 March 2014 and expiry date 30 September 2015) that contained type O virus antigen was used because this strain is active in Cambodia. The adjuvant was double oil emulsion (DOE: water‐in‐oil‐in‐water) and the recommended storage temperature range for this vaccine was 2–8°C. FMD vaccines were stored in a refrigerator until just prior to use in the experiment when they were treated for 7 days by placing the vaccine in a freezer, refrigerator or leaving it at the ambient indoor temperature. The storage temperatures during this period were monitored using data loggers (Thermochron^®^ DS1921, Dallas Identification/ALFA‐TEK, Bayswater, Victoria, Australia) programmed to read temperatures at 5‐minute intervals with an accuracy of ±1°C. The same procedure was undertaken prior to the day 30 booster vaccination with each animal boosted with vaccine given the same temperature treatment as the primary vaccination.

### Vaccination and blood sampling procedure

Cattle were vaccinated with a full (2 mL, FD) or half (1 mL, HD) dose of the treated vaccines on day 0 with one animal on each farm receiving each of the six treatment combinations (Freezer FD, Freezer HD, Fridge FD, Fridge HD, Ambient FD, Ambient HD). All cattle were reported to be naive to FMD vaccination for the last 3 years and the six vaccination treatments were applied to cattle at random within each farm. Booster vaccination (first re‐vaccination) with vaccine of the same treatment and dose was 30 days later (30 dpv) as recommended by the vaccine manufacturer. A full dose of properly stored current vaccine was administrated after the third blood sampling at 60 dpv to hopefully provide full protective immunity. Blood for serum was collected before vaccine administration at 0, 30 and 60 dpv. Farmers were informed of a visit the day before and tethered the selected cattle. A simple crush was set up by an experienced village animal health worker (VAHW) and staff of the Provincial Office of Animal Health and Production (POAHP) with assistance from the farmer. Both vaccination and blood collection were conducted by a single experienced operator to remove operator bias. Vaccines were administered subcutaneously in the neck region and blood was collected directly into 5 mL vacutainer tubes by jugular venipuncture. After collection, blood was allowed to clot, then centrifuged and serum transferred into a new Eppendorf tube and stored at −20°C until required.

### Serological tests for FMD antibody detection

A total of 315 sera were collected during the study with nine animals not available at the final sampling due to failure to return from the forest (8) or death due to a road accident (1). Samples were subjected to a non‐structural protein ELISA (NSPE) and liquid‐phase blocking ELISA (LPBE) for structural protein at National Veterinary Research Institute (NaVRI) as shown in Table 2. The NSPE detects antibodies to viral non‐structural proteins indicating present or past active infection with FMDV (VDPro^®^ ELISA Diagnostics FMDV, MEDIAN Diagnostics Inc.) (Sørensen *et al*. 1998; Bergmann *et al*. 2000; Brocchi *et al*. 2006). The LPBE detects antibodies directed against structural FMD proteins elicited by both vaccination and natural infection (Hamblin *et al*. 1986; Alexandersen *et al*. 2003b). The reagents for LPBE were supplied by the Regional Reference Laboratory, Pakchong, Thailand and prepared according to their guidelines. For the NSPE results, the sera were considered positive at optical density (OD) <0.6 while in LPBE the sera with titres less than 1/40 (Log_10_ 1.6) were considered to be negative(Hamblin *et al*. 1987; OIE, 2012).

**Table 2:** Number of NSPE and LPBE serological tests performed

Time post initial vaccination (day)	NSPE (3ABC)	LPBE	Total
0	108	108	216
30	No test	108	108
60	a99	99	198
Total	207	315	522

a Nine cattle were not available on the day 60 sample collection.

### Statistical analysis

All data were collated on an individual animal basis and analysed using JMP 12 (SAS Institute Inc., NC, USA). For NSPE results, the proportion of test positives at each sampling was analysed using contingency table analysis (Pearson Chi square test). For the LPBE results, the log_10_ LPBE titres at each sampling were analysed fitting the effects of village, NSP result at day 0 (positive/negative), LPBE titres at day 0, sex, body condition score, previous FMD infection and treatment effects (dose, storage temperature and the interactions between them). The significance of differences between means within a significant effect was determined by fitting orthogonal contrasts within the model, or by Turkey's HSD test. Results of analyses are presented as least squares means with standard errors and a significance level of *P* < 0.05 is used throughout.

## Results

### Application of treatments

Information from the temperature data loggers on treated FMD vaccines used for the day 0 and 30 vaccinations is summarised in Table 3. There were no reports of new cases of FMD from any of the participating villages during the course of the experiment.

**Table 3:** The results of FMD vaccine treatment temperatures recorded by data loggers (all figures in °C)

Time of vaccination	Freezer			Refrigerator			Ambient		
	Mean	SE	Min ‐ Max	Mean	SE	Min ‐ Max	Mean	SE	Min ‐ Max
Day 0	−13.3	0.024	−15 to −1	5.3	0.012	4 to 7	30.1	0.024	28 to 32
Day 30	−14.5	0.043	−17 to −1	2.7	0.010	2 to 5	27.2	0.025	25 to 30

### Natural infection‐specific ELISA (NSPE)

The number of seropositive cattle at day 0 and 60 by village is summarised in Table 4. The results of the NSPE revealed that only 5/108 (4.6%) cattle on two farms were seropositive at the commencement of the study indicating prior infection with FMD. By day 60, the remaining four of these five animals remained positive and a further 15 animals had seroconverted resulting in 19/99 positives (19.2%) on 10 farms, a significant increase (*P* = 0.001). The incidence of positives at day 60 was markedly higher in older cattle with 0/27 (0%), 5/35 (12.5%) and 14/32 (43.8%) of animals age classes 1 year or less, 1‐3 years and >3 years, respectively (*P* < 0.0001).

**Table 4:** The results of NSPE on days 0 and 60 post initial FMD vaccination

Village	Day 0			Day 60		
	No. of cattle tested	No. positive	Positive (%)	No. of cattle tested	No. positive	Positive (%)
Prey Ourmal	36	0/36	0.0	35	7/35	20.0
Roleap	36	2/36	5.6	28	6/28	21.4
Or Thkov	36	3/36	8.3	36	6/36	16.7
Total	108	5/108	4.6	99	19/99	19.2

Some farmers indicated that they experienced possible FMD infection (blisters in the mouth and on feet) in the past. Of the 108 cattle, 12 from two farmers in Or Thkov village and two from a farmer in Prey Ourmal village were reported as sick in 2011 and 2013, respectively. Only three of those 14 were seropositive for FMD on day 0. However, the number of seropositive in this group increased from 3 at day 0 to 7 at the day 60.

### Liquid‐Phase Blocking ELISA detecting vaccination and natural infection

On day 0, 79/108 (73.2%) samples were negative for the LPBE test with the 29 positive samples including the five samples positive to the NSPE test. By day 30, 103/108 (95.4%) samples were positive and by day 60 99/99 (100%) of samples were positive (Table 5) indicating high levels of vaccination response at days 30 and 60. There were no significant treatments effects on the proportion of positive samples.

**Table 5:** Proportion of samples falling into different LPBE antibody titre categories by days post primary vaccination, vaccine storage treatment and vaccine dose

Proportion of total vaccinated animals with different LPBE antibody titres																		
LPBE titre	Day 0						Day 30						Day 60					
	Freeze		Fridge		Ambient		Freeze		Fridge		Ambient		Freeze		Fridge		Ambient	
	HD	FD	HD	FD	HD	FD	HD	FD	HD	FD	HD	FD	HD	FD	HD	FD	HD	FD
0	3/18	2/18	1/18	6/18	3/18	3/18	0/18	0/18	0/18	0/18	0/18	0/18	0/15	0/17	0/17	0/17	0/16	0/17
40	7/18	14/18	12/18	9/18	9/18	10/18	1/18	0/18	2/18	0/18	2/18	0/18	0/15	0/17	0/17	0/17	0/16	0/17
80	5/18	2/18	3/18	3/18	2/18	2/18	1/18	5/18	3/18	0/18	1/18	3/18	0/15	0/17	0/17	0/17	1/16	0/17
160	3/18	0/18	2/18	0/18	3/18	2/18	5/18	6/18	3/18	8/18	5/18	5/18	1/15	0/17	0/17	0/17	0/16	0/17
320	0/18	0/18	0/18	0/18	1/18	1/18	10/18	5/18	8/18	9/18	8/18	6/18	1/15	2/17	2/17	0/17	2/16	0/17
640	0/18	0/18	0/18	0/18	0/18	0/18	1/18	2/18	1/18	0/18	1/18	4/18	5/15	6/17	5/17	4/17	3/16	1/17
1,280	0/18	0/18	0/18	0/18	0/18	0/18	0/18	0/18	1/18	1/18	1/18	0/18	2/15	5/17	4/17	3/17	2/16	4/17
2,560	0/18	0/18	0/18	0/18	0/18	0/18	0/18	0/18	0/18	0/18	0/18	0/18	4/15	4/17	4/17	5/17	7/16	9/17
5,120	0/18	0/18	0/18	0/18	0/18	0/18	0/18	0/18	0/18	0/18	0/18	0/18	2/15	0/17	2/17	5/17	1/16	3/17
Total positive	8/18	2/18	5/18	3/18	6/18	5/18	17/18	18/18	16/18	18/18	16/18	18/18	15/15	17/17	17/17	17/17	16/16	17/17

*Note:* FD=full dose and HD=half dose.

Analysis of LPBE antibody titre showed that overall Log_10_ titres increased steadily from day 0 (1.46 ± 0.07) to day 30 (2.34 ± 0.03) and 60 (3.15 ± 0.04) of the experiment. Mean levels exceeded the protective level of 1.8 from day 30 onwards. At day 0, titres were low with significant effects of village (*P* = 0.012) and age class (*P* = 0.07). Or Thkov village had significantly higher titres than the Prey Ourmal with Roleap intermediate. Animals older than 3 years of age had significantly higher titres (1.86 ± 0.20) than those under 1 year of age (1.20 ± 1.18) with animals aged between 1 and 3 years intermediate (1.60 ± 0.21). The effects of initial NSP titre, sex, body condition score and previous illness status were all non‐significant.

**Figure 1:**
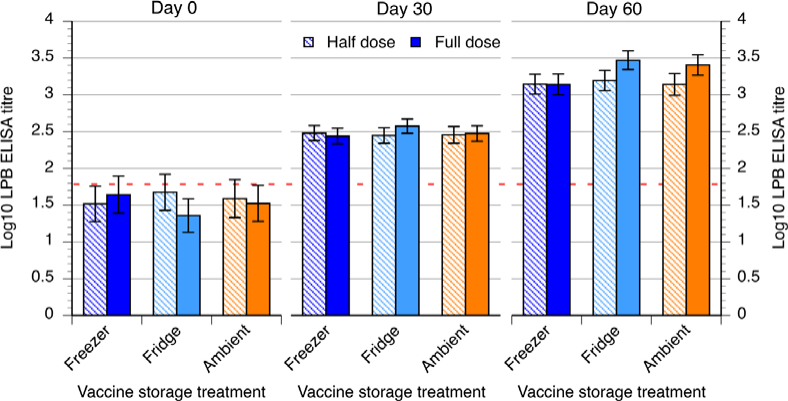
Log_10_ LPB ELISA titres (LSM±SE) at 0, 30 and 60 days post primary vaccination showing the effects of vaccine storage treatment and vaccine dose. Half dose is represented by the striped pattern while the full dose is represented by the solid colour pattern. The dotted red line is represented the ELISA threshold for the protective titre.

Analysis of titres at day 30 revealed significant effects of village (*P* = 0.002) and initial LPBE titre at day 0 (*P* = 0.001), but not vaccine storage temperature (*P* = 0.691) or dose (*P* = 0.530, Fig. 1). None of the other effects or interactions was significant. The village Roleap had significantly lower titres than the other two villages (*P* = 0.002) and there was a positive association between Log_10_ LPBE titre at day 0 and day 30 (*P* = 0.001).

Analysis of titres at day 60 again revealed significant effects of village (*P* = 0.01) and initial LPBE titre at day 0 (*P* = 0.007). The effect of vaccine dose (*P* = 0.02) was also significant and there was a trend towards an effect of vaccine storage temperature (*P* = 0.094, Fig. 1). The effects of initial NSP titre, sex, age class, and body condition score were all non‐significant. By day 60, Othkov village had the lowest average Log_10_ LPBE antibody titre (3.08 ± 0.11) with Roleap (3.30 ± 0.13) intermediate between it and Prey Ourmal (3.37 ± 0.13). As at day 30, a positive association between initial LPBE titre and that day 60 was found (*P* = 0.007). Cattle receiving a full dose of vaccine had higher titres at day 60 than those receiving a half dose (3.34 ± 0.11 vs. 3.16 ± 0.12, *P* = 0.02) and there was trend (*P* = 0.094) towards lower titres in animals receiving frozen vaccine (3.14 ± 0.12) than that stored in the refrigerator (3.33 ± 0.11) or at ambient temperatures (3.27 ± 0.13) for 7 days. The specific contrast between the titres of animals given frozen vaccine against the other two treatments combined was significant (*P* = 0.039) indicating a significant negative effect of freezing vaccine for 7 days (Fig. 1).

## Discussion

FMD is endemic in Cambodia as well as many other countries in Southeast Asia. Over the last 10 years, Serotype O has been the common serotype detected in outbreaks there. In Cambodia, the major control strategy for FMD is vaccination. The aim of this study was to determine whether inappropriate storage temperatures and/or under dosing will reduce vaccine efficacy in the field. The results showed that using a half dose rather a full dose, and freezing of vaccine for a week prior to administration significantly reduced the antibody response to vaccine 30 days after a primary and booster vaccination whereas exposure to ambient temperatures had no effect. However, the reduction in titre did not translate into decline below the effective threshold.

At the commencement of the study, the infection‐specific NSPE test indicated that 4.6% of experimental cattle had prior FMD infection, while 26.8% were positive to the LPBE test which detects exposure to both active and killed vaccine. This indicates relatively low levels of natural exposure and vaccination in the test population.

There was a clear response to both the primary and booster vaccinations with all treatments achieving mean antibody responses above acknowledged protective Log_10_ LPBE value of 1.6 (Fig. 1). The early protective response is consistent with results of Golde *et al*. (2005) and Doel *et al*. (1994) who reported that FMD vaccination can fully protect cattle against direct challenge with virus as little as 7 and 4 days later, respectively. The significant protective response in all treatments showed that the vaccine treatments did not abolish efficacy. This is consistent with the finding of El‐Sayed *et al*. (2012) that oil adjuvant inactivated FMD vaccine maintained its potency for 3 weeks when stored at a temperature of 25°C. Butchaiah *et al*. (1985) also showed that a saponified aluminium hydroxide gel‐adsorbed, formaldehyde‐inactivated FMD vaccine keep its potency for at least 2 days at 25°C. Our own findings were that ambient temperatures well above the recommended storage temperature for 1 week had no effect on antibody response to vaccination are consistent with this. However, freezing for 1 week did have a deleterious effect on the antibody response to vaccination, while not ablating the response. In a recently reported study of 30 vaccine storage sites in Cambodia in which temperatures were monitored hourly for 1 month, we found that 15.4% of temperature readings were below freezing, whereas 18.5% were above 8°C (Sieng *et al*. 2016). This indicates that vaccine freezing is a significant risk during storage.

While the study results are encouraging because they suggest that relatively short aberrations in storage conditions are unlikely to influence vaccine efficacy greatly, it must be noted that this experiment did not test longer term exposure to non‐recommended temperatures, nor did it test the effect of shorter term deviations on the stability of the vaccine and duration of efficacy, even if stored at recommended temperatures following or between the deviations. Halving the dose of vaccine administered significantly reduced the antibody response to vaccination demonstrating clearly that dose cutting is likely to contribute to vaccine failure. Similar results were obtained from an experiment with different doses of high potency FMD vaccine (full dose, 1/4 dose and 1/16 dose of vaccine) in which level of protection against FMD was decreased when vaccine dose was reduced (Brehm *et al*. 2008). Cortese & Smith (2004) also reported reduced immune response and increased risk of future anaphylactic reactions when partial dosing was used.

The increase in NSPE titres between day 0 and 60 of the experiment was an unexpected finding and suggestive of an increased infection rate of animals with FMD during the experiment. However, there was no reported incidence of FMD during the 60 days of the study in the study animals or the villages they inhabited. It may be that the rapid protective effects of vaccination, as discussed above obscured any new infection. It remains unknown whether the increase was due to a natural increase in FMD infection during the period, whether the experiment itself facilitated transmission, or whether the NSPE test for day 60 showed greater sensitivity than the sample run for the day 0 samples. Overall, these results have shown that typical perturbations in FMD vaccine storage temperatures for up to a week have comparatively minor but significant immediate effects on vaccine efficacy. Freezing vaccine with associated thawing rather than storage at ambient temperatures had an adverse effect, as did using a half dose of vaccine. It is recommended that freezing be avoided as should routine administration of half doses. In order to retain maximum potency vaccines should be stored at the recommended temperature range (2–8°C) whenever possible (Butchaiah *et al*. 1985; El‐Sayed *et al*. 2012), and a full dose be administered.

## Source of funding

This study was supported by the Australian Centre for International Agricultural Research, through Project AH/2010/046 and by the General Directorate of Animal Health and Production, Ministry of Agriculture, Forestry and Fisheries, Government of the Kingdom of Cambodia.

## Conflicts of interest

The authors declare that they have no conflicts of interest.

## Ethics statement

The authors confirm that the work complies with the ethical policies of the journal. The work was approved by the National Veterinary Research Institute of the General Directorate of Animal Health and Production in Cambodia and by the Animal Ethics Committee of the University of New England (Armidale, Australia) under Authority No.: AEC 13‐125.

## Contributions

The authors were involved in the concept and design of the study as well as part of the collection process, analysis and interpretation of data. All authors contributed critically to revising the manuscript and had read and approved the final version.
